# Understanding Individual Differences in Metacognitive Strategy Use, Task Demand, and Performance in Integrated L2 Speaking Assessment Tasks

**DOI:** 10.3389/fpsyg.2022.876208

**Published:** 2022-06-14

**Authors:** Weiwei Zhang, Meijuan Zhao, Ye Zhu

**Affiliations:** ^1^School of Foreign Languages and International Education, Quzhou University, Quzhou, China; ^2^Graduate Institute of Interpretation and Translation, Shanghai International Studies University, Shanghai, China; ^3^Institute of Linguistics, Shanghai International Studies University, China Center for Language Planning and Policy Studies, State Language Commission of China, Shanghai, China

**Keywords:** individual differences in metacognitive strategy use, task demand, speaking performance, integrated L2 speaking assessment tasks, Kormos’ Bilingual Speech Production Model

## Abstract

This study investigated the concept of individual differences (IDs) in the use of metacognitive strategies (planning, problem-solving, monitoring, and evaluating) and its relationship with task demand and learner performance within Kormos’ Bilingual Speech Production Model from the lens of Chinese English-as-foreign-language (EFL) learners in the context of integrated L2 speaking assessment. To measure metacognitive strategies, we administered an inventory on 134 Chinese EFL learners after they completed four integrated L2 speaking assessment tasks. Descriptive analysis and multiple linear regression were adopted for data analysis, and results show that: (a) IDs displayed variance in Chinese EFL learners’ metacognitive strategy use; (b) among the four metacognitive strategies under investigation, problem-solving was reported to be used the most frequently in sharp contrast to monitoring, which had the lowest frequency; (c) metacognitive strategies worked interactively, responding to task demands involved in the four integrated L2 speaking assessment tasks; and (d) Chinese EFL learners’ use of metacognitive strategies, in individual and interactive working modes, had no relationship with their speaking performance. These results are expected to present some insights into the role of IDs in metacognitive strategy use during L2 speech production under assessment conditions, which will add robust evidence to the existing literature on L2 speaking, in particular on metacognitive strategy use in L2 speaking assessment. In the meantime, the findings will provide some empirical validation support for Kormos’ model, which will further provide some implications for L2 speaking instruction and L2 assessment.

## Introduction

In the four language skills (reading, listening, writing, and speaking), speaking is acknowledged as the most intricate productive skill to master, and speaking in a foreign language is even more complicated in that speaking is done in real-time, imposing heavy demands on speakers’ abilities to use metacognitive strategies, core individual differences (IDs) construct (e.g., Luoma, 2004; Yahya, 2019; Newton and Nation, 2020; Sun, 2020; Griffiths and Soruç, 2021). Therefore, speaking, among the four language skills, has been proposed to have the closest relationship with foreign and/or second language (L2) speakers’ IDs in metacognitive strategy use, empowering L2 speakers to plan the knowledge at hand and to compensate for and facilitate their oral production so as to affect their ultimate speaking performance (Kormos, 2006, 2011; Bygate, 2011; Cohen, 2014). Nonetheless, such a salient role of IDs in metacognitive strategy use in L2 speaking has not been paid sufficient attention, and the available literature along this line of research inquiry primarily focuses on how L2 speakers use metacognitive strategies in non-assessment contexts (Zhang et al., 2021a). Consequently, how IDs in metacognitive strategy use functions in L2 speaking assessment for a smooth speech production still remains unclear, which rationalizes the research context of L2 speaking assessment in our study.

Additionally, there is extensive acknowledgment of the backwash effect of L2 assessment on L2 learning, and an ever-increasing recognition of adopting a holistic approach in L2 instruction through the use of integrated skill tasks involving multiple language skills to familiarize learners with authentic language use tasks for improving their language ability (Newton and Nation, 2020). As such, in formulating the research context of L2 speaking assessment, we embedded our study within the specific context of integrated L2 speaking assessment that involves not only speaking but listening and reading as well.

In the extant literature, although inconsistency still exists in the conceptualization of metacognitive strategies, an agreement has been reached among scholars (e.g., Cohen, 2014; Oxford, 2017) that studies into this concept should be contextualized in accordance with the specific language skill under investigation. Hence, to conceptualize the IDs construct of metacognitive strategies in L2 speaking, we framed our study within Kormos’ (2006, 2011) Bilingual Speech Production Model (hereinafter referred to as Kormos’ Model where necessary), a model that has been recognized as authentically duplicating the operating mode of metacognitive strategies in L2 speech production (Skehan, 2016, 2018). Furthermore, Kormos’ Model has been widely applied in empirical studies on L2 speaking as the major Bilingual Speech Production Model (e.g., Kormos, 2011; Yahya, 2019) and has been accredited as “more elaborate and more targeted” (Wang and Liu, 2018, p. 397), compared with other L2 speech models, due to its solid theoretical grounding and strong empirical support (Wang and Liu, 2018; Yahya, 2019).

Taken together, we investigated IDs in metacognitive strategy use within Kormos’ Model in the context of integrated L2 speaking assessment. In the research field of IDs, it is considered that IDs variables, including metacognitive strategies, interact with external factors such as context and tasks, affecting learner performance (e.g., Griffiths and Soruç, 2021). By the same token, in Kormos’ Model, metacognitive strategies are also proposed to work, in independent and interactive manners, with tasks, exerting influence on performance. In line with this, to comprehensively study IDs in metacognitive strategy use in our research context, we set our focus on not only the concept *per se* but also its relationship with task and performance as well.

The novelty of our study is to add empirical evidence to the existing literature on IDs in metacognitive strategy use in L2 speech production under assessment conditions, while providing validation support for Kormos’ (2006, 2011) model. Simultaneously, our study is expected to enrich the understanding of integrated L2 speaking assessment, an under-explored field (Frost et al., 2020; Zhang et al., 2021a). Moreover, the study is hoped to offer some implications for L2 speaking instruction, in particular, metacognitive instruction on L2 speaking and L2 assessment.

## Literature Review

### Individual Differences in Metacognitive Strategy Use

Evolving from the research domain of second language acquisition, the concept of individual differences (IDs) has developed into a formal field of scholarship, contributing to a veritable plethora of literature. Despite this, consensus on the taxonomies of IDs is far from absolute (e.g., Griffiths and Soruç, 2021). In a most recent publication, Griffiths and Soruç (2021) defined this concept as “characteristics which make learners different from each other and which affect the way that they behave in the classroom and beyond” (p. 341) based on their extensive review of the literature on IDs. They further proposed 11 learner variables (*viz*. motivation, aptitude, strategies, gender, culture/nationality/ethnicity/race, beliefs, autonomy, personality, style, age and affect) that attribute to IDs in terms of affecting language learning and teaching based on the findings of their empirical study, among which, strategies, especially metacognitive strategies, and motivation have a stronger influence on IDs in comparison with other variables. In addition to Griffiths and Soruç (2021), a large volume of literature has also evidenced the role of metacognitive strategies as a contributing variable to IDs, and hence the concept of metacognitive strategies is also termed IDs construct in the research arena of IDs (e.g., Oxford and Amerstorfer, 2018; Psaltou-Joycey and Gavriilidou, 2018). In accordance with this term, we labeled learners’ use of metacognitive strategies as IDs in metacognitive strategy use in this study as shown throughout.

Metacognitive strategies originate from the field of psychology as a pivotal element of metacognition, a concept coined by Flavell (1979) that “refers to one’s knowledge concerning one’s own cognitive process and products or anything related to them” (p. 32). Since metacognition is multi-faceted, “multidimensional and domain-general in nature” (Teng et al., 2021a, p. 169), a consistent debate has been existing around the definition and components of the concept during its evolution (e.g., Zhang et al., 2021b). Regardless, it is acknowledged that the foundational research on metacognition takes root in two frameworks proposed by Flavell (1979) and Brown (1987) (Sperling et al., 2012; Nazarieh, 2016), in which metacognition is agreed to be comprised of metacognitive knowledge encompassing person/declarative knowledge, task/procedure knowledge and strategy/conditional knowledge, and metacognitive regulation or metacognitive strategies composed of planning (planning individual’s learning activities in accordance with their learning objectives prior to L2 learning), monitoring (online monitoring in the individuals’ learning process) and evaluating (post-learning evaluating of the learning process) with the three components working independently and interactively (refer to Teng et al., 2021b; Zhang, 2021, for a review). In the most updated model of metacognition, though focusing on writing, established by Teng et al. (2021a), metacognitive strategies are also proposed to comprise three key components: planning, monitoring, and evaluating.

Due to the crucial role of metacognition in language learning and teaching (e.g., Oxford, 2017), metacognitive strategies are also recognized as one form of language learning strategies (LLSs) and have been reported to be the most important LLSs in a learner’s successful learning (Zhang and Zhang, 2018; Gan et al., 2020). Like metacognition, the trajectory of metacognitive strategies is also characterized by debate on the concept’s definition and taxonomies in the field of LLSs, which has been manifested by various models, including the widely applied Oxford’s (1990) Strategy System Model of Learning Strategies and O’Malley and Chamot’s (1990) Strategy Taxonomy Model. In spite of their seeming differences, all these models “reflect relatively the same categorizations of language learning strategies without any fundamental changes” (Zare, 2012, p. 164), and the key elements of metacognitive strategies across these models are consistent: planning, problem-solving, monitoring, and evaluating. Planning refers to L2 learners’ learning activities for achieving their learning goals before L2 learning; problem-solving implies the employment of various methods to solve learning problems such as substitution, inferencing, and the use of gap fillers; monitoring denotes L2 learners’ online inspection of their learning process; and evaluating images learners’ post-learning assessment of their learning process (refer to Zhang, 2021, for an overview). The four metacognitive strategies operate independently and interactively to influence performance through their interactions with external tasks.

It is obvious that there is a great overlap between the research field of metacognition and the LLSs in terms of the components and the working mode of metacognitive strategies (An and Gan, 2021). In fact, such an overlap is also manifested in L2 speech production within Kormos’ Model as reviewed in the subsequent section.

### Conceptualizing Individual Differences in Metacognitive Strategy Use in Kormos’ Model

As stated earlier (refer to “INTRODUCTION” section), the conceptualization of IDs in metacognitive strategy use during L2 speech production in our study was conducted through framing the concept in Kormos’ Model. In the research field of speaking, models generated in psycholinguistics are broadly acknowledged and employed (e.g., Kormos, 2006, 2011; Skehan, 2016; Yahya, 2019; Sun, 2022a,b), among which Levelt’s (1989) model of monolingual speech production has become “one of the most comprehensive and widely used theoretical frameworks” for monolingual speech production (Sun, 2016, p. 27). Based on this model, De Bot (1992) proposed his L2 speech production model, followed by many similar research efforts (e.g., Poulisse and Bongaerts, 1994; Towell et al., 1996). More recently, integrating Levelt’s (1989) L1 model and existing L2 speech models, Kormos (2006, 2011) mapped out the Bilingual Speech Production Model, a substantially influential bilingual speech model employed in L2 speaking studies (Wang and Liu, 2018).

Kormos’ Model is modular, and it consists of separate encoding modules: a conceptualizer for planning the message, a formulator for linguistically encoding the message, and an articulator for articulating the encoded message as sounds. In addition, the model also encompasses a large knowledge store labeled as long-term memory which comprises elements such as lexicon and syllabary that provide L2 speakers with the information needed; a speech comprehension system receives L2 speakers’ actual discourse for inspection *via* monitoring, and an audition component (an acoustic-phonetic processor) helps the monitor to check the produced utterance. The monitor is based on the conceptualizer, monitoring the outputs of the conceptualizer, the formulator, and the whole process of speech production.

In correspondence to the four modules are the four stages in L2 speech production. They include: conceptualization in which speakers plan, at macro- and microlevels, what to speak or the intended message; formulation where speakers encode linguistically the intended message; articulation through which speakers execute their speech sounds by controlling the articulatory muscles, converting the phonetic plan generated in formulation to overt speech; and monitoring with which speakers check and notice errors for possible modifications and corrections to make their utterance in light of the task demands. Although the role of evaluating is not explicitly emphasized in the model, it should be noted that during monitoring in the different stages of L2 speech production, speakers are assumed to use evaluating in tandem with monitoring (O’Malley and Chamot, 1990) because without evaluating, L2 speakers are unlikely to make comparisons between the preverbal plan produced in conceptualization and the intended messages encoded in the formulation. Similarly, when L2 speakers use monitoring to examine the internal speech in the formulation and the overt speech in articulation, they have to use evaluation; otherwise, they cannot judge whether or not their actual utterances are consistent with task demands (Purpura, 1999). In other words, evaluation plays an equally important part as monitoring in L2 speech production (Zhang, 2021).

In L2 speech production, since speakers’ L2 knowledge may not be complete, it is unavoidable that they will encounter problems (Dörnyei and Kormos, 1998), and how speakers solve these problems is demonstrated by problem-solving mechanisms in Kormos’ Model. According to Kormos (2006, 2011), there are four types of problems that L2 speakers may tackle in speech production. The first type is resource deficit which normally occurs in conceptualization and formulation, relating to L2 speakers’ language knowledge gap that prevents them to verbalize their intended messages. For problems of this type, solutions include substitution. Time pressure is another type of unavoidable problem that L2 speakers frequently encounter in planning and processing their speech. Solutions to the problem are pauses and repetitions, such as the use of gap fillers. The third type of problem is perceived deficiencies in L2 speakers’ language output displayed by the incorrectness or inappropriateness of their utterances, and relevant solutions are self-repair and self-appraising. Finally, the fourth type of problems is the perceived deficiencies in the interlocutor’s performance, which are commonly solved by L2 speakers through the use of communicative strategies immediately related to metacognitive strategies (e.g., Bachman and Palmer, 2010; Ellis et al., 2019). It is evident that all the problem-solving mechanisms operating in Kormos’ Model essentially replicate L2 speakers’ employment of the metacognitive strategy of problem-solving as delineated earlier.

To summarize, the IDs construct of metacognitive strategies works in the forms of planning, problem-solving, monitoring, and evaluating individually and interactively, affecting L2 learners’ speaking performance in Kormos’ Model. Integrating the taxonomies and working mode of metacognitive strategies in this model with those in the literature on metacognition and LLSs as reviewed above, we conceptualized the IDs construct of metacognitive strategies as four metacognitive strategies: planning, problem-solving, monitoring, and evaluating, and accordingly IDs in metacognitive strategy use are conceptualized as individuals’ use of the four metacognitive strategies which function independently and interactively, responding to task demand and impacting performance.

### Integrated L2 Speaking Assessment Tasks: Task Demand

Integrated L2 speaking assessment tasks stand for a comparatively new and dynamic assessment/testing format that integrates reading, listening, and speaking to measure L2 learners’ speaking performance (Frost et al., 2020). In comparison with other L2 tasks, this type of tasks is closer to real-world speaking tasks, which normally require speakers either to listen or read or to listen and read before speaking. It is agreed that integrated L2 speaking assessment tasks elicit a broad range of strategies from L2 speakers and have an intimate relationship with learners’ use of metacognitive strategies (Barkaoui et al., 2013; Cohen, 2014). The close connections to real oral communications have made integrated L2 speaking assessment tasks ideal tasks for L2 speaking classroom instruction (Zhang et al., 2021a,b). Regardless, limited attention has been devoted to this test format in research actuality, which also accounts for the contextualization of our study as noted previously. In the available studies on integrated L2 speaking assessment tasks, the common practice is that researchers adopted the test of English as a foreign language (TOEFL) iBT integrated speaking section composed of four tasks that involve varying degrees of task demand (Barkaoui et al., 2013). Following the spirit of these researchers, we also contextualized our research in this test which concomitantly serves as one of the instruments in this study as described later.

**FIGURE 1:**
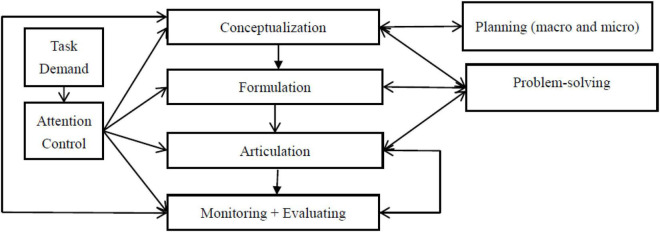
Working mode of metacognitive strategies in Kormos’ Model.

In Kormos’ Model, the four TOEFL iBT integrated speaking tasks are proposed to influence L2 speakers’ purposeful use of metacognitive strategies on the grounds that conceptualization, formulation, and monitoring are subject to L2 speaker’s conscious attentional control determined by task demands. As pointed out by Kormos, an individual’s attention resources are limited; hence, the three stages in L2 speech production naturally compete with one another for the attention available. How the limited attention is allocated among the three stages is considerably impacted by task demands. For instance, when task demands are increased, L2 speakers are expected to allocate increasing attention to analyze task characteristics and to plan the conceptualization. As a result, a more complex preverbal plan may be generated. To encode the plan with increased task demands from the perspective of linguistics, L2 speakers are very likely to invest more attention in formulation. After L2 speakers consciously increase the amount of their attention to conceptualization and formulation, the attentional resources controlled by these speakers for monitoring and evaluating will be accordingly reduced, which indicates that more errors may be undetected in various stages of speech production, including the speakers’ final speech. Hence, the quality of the speakers’ performance will be negatively affected. The working mode of the four metacognitive strategies subject to attentional control caused by task demand variability in Kormos’ Model is illustrated in Figure 1, which essentially illustrates our study framed in the model.

### Empirical Studies

As stated earlier, studies on IDs in metacognitive strategy use and its effect on learner performance contextualized in L2 speaking assessment are quite limited. In these studies, those that were conducted in the specific context of integrated L2 speaking assessment are even fewer. To our knowledge, most of the current literature on IDs in metacognitive strategy use has focused either on the relationship between the concept and performance contextualized in the other three language skills (e.g., listening by Nett et al., 2012) and the relationship between tasks and performance (e.g., Rukthong and Brunfaut, 2020), or on the relationship between the concept, tasks, and performance in the context of independent speaking tests (e.g., Fernandez, 2018). In fact, there are only three studies, Swain et al. (2009), Yi (2012), and Barkaoui et al. (2013), in the literature that have investigated the intricate relationship between IDs in metacognitive strategy use, task demand, and performance contextualized in integrated L2 speaking assessment, as was the case with the present study.

In an exploratory approach, Swain et al. (2009) investigated 30 Chinese EFL learners’ metacognitive strategy use in processing the TOEFL iBT integrated speaking tasks and its relationship with their speaking performance reflected by their test scores. The study showed that the participants frequently used metacognitive strategies, and there was no direct relationship between metacognitive strategy use and speaking performance. Barkaoui et al. (2013) re-conducted this study and reached similar findings. In the same research methodology as in Swain et al.’s (2009), Yi (2012) collected data on six Korean EFL university students’ metacognitive strategy use and test scores in performing TOEFL-based speaking test tasks in both testing and non-testing conditions. The subsequent data analysis disclosed that the participants used metacognitive strategies frequently, and a weak relationship between metacognitive strategy use and speaking performance under both testing and no-testing conditions was founded.

However, as Zhang (2021) has pointed out, none of these studies are without limitations, and this indicates the research gaps that we aimed to fill in this study. First, researchers collected data on a small sample (no more than 30), which places the validity and generalizability of the research findings into question (Creswell and Creswell, 2018). Second, since a study into metacognitive strategies is proposed to be contextualized in accordance with the language skill it intends to investigate as noted afore (Cohen, 2014; Oxford, 2017), the exploratory approach to researching L2 learners’ metacognitive strategy use deployed in these studies suggests a lack of focus on the IDs construct operating specifically in L2 speech production where metacognitive strategies work in the form of planning, problem-solving, monitoring, and evaluating as delineated in the above literature review. Last but not the least, metacognitive strategies used by the participants in the studies were investigated in an individual manner, and the interactions within the components of metacognitive strategies as well as their response to task demands were not examined, which was not consistent with either the working principle of IDs in metacognitive strategy use or the working mode of metacognitive strategies as reviewed earlier.

## Methodology

### Research Questions

To fill the above research gaps, built upon our review of the literature, our study addressed the following research questions (RQ) through an investigation into a rather large sample size formulated by 134 Chinese EFL learners, and our examination of IDs in metacognitive strategy use covered both the independent and the interactive aspects of the working mode of metacognitive strategies:

**RQ1**: How do Chinese EFL learners’ IDs in metacognitive strategy use work in the context of integrated L2 speaking assessment tasks within Kormos’ Model?

**RQ2:** What are the relationships among Chinese EFL learners’ IDs in metacognitive strategy use, task demand, and their performance in the context of integrated L2 speaking assessment tasks within Kormos’ Model?

### Participants

As noted above, our study involved a total of 134 Chinese EFL learners by means of convenience sampling. On a voluntary basis, the participants came from two universities situated on the Mainland of the People’s Republic of China. The percentages of male and female students are 38 and 62%, and their age range was 18–21 years. All the participants have passed College English Test—Band 4 (CET-4), an authoritative English language proficiency test with high reliability administered specifically to university students in China, which, to a great extent, guarantees the participants’ language proficiency to perform the four TOEFL iBT integrated speaking tasks for the smooth progress of this study (Zhang, 2021). Two trained raters, who had the experience in rating the TOEFL iBT integrated speaking section, scored the Chinese EFL learners’ speaking performance.

### Instruments

#### The Strategic Competence Inventory for Computer-Assisted Speaking Assessment

We deployed the Mandarin Chinese version of Zhang et al.’s (2021a) Strategic Competence Inventory for Computer-Assisted Speaking Assessment or SCICASA to measure the metacognitive strategies used by Chinese EFL learners in our study. The rationales for doing so are as follows: (a) Our research context, the TOEFL iBT integrated speaking tasks, is one form of computer-assisted speaking assessment. (b) The four operating forms of IDs in metacognitive strategy use under investigation are planning, problem-solving, monitoring, and evaluating, which are consistent with the four dimensions of the strategic competence in the inventory. (c) Inventories are widely applied in exploring L2 learners’ internal metacognitive activities. (d) The native language of the Chinese EFL learners in our study is Mandarin Chinese.

The SCICASA has high validity and reliability (α = 0.941), and it has 23 items classified into four dimensions: planning, problem-solving, monitoring, and evaluating. Five structured questions on L2 learners’ background information (e.g., age, gender, and language proficiency) are also included in the inventory. A 6-point Likert scale is used for each item: 0 (never or almost never), 1 (rarely), 2 (sometimes), 3 (often), 4 (usually), and 5 (always or almost always) (refer to Zhang et al., 2021a, for the detailed documentation of the inventory).

#### TOEFL iBT Integrated Speaking Tasks

The TOEFL iBT integrated speaking section served both as the research context of our study and as the instrument that was used to elicit Chinese EFL learners’ speaking performance, as noted previously. Because our study was conducted in 2018 before the most recent reform in TOEFL iBT integrated speaking section that took place in 2019, the speaking section that we selected came from the old version of this test. The section comprised of four tasks (Task 1, Task 2, Task 3, and Task 4) that involve varying degrees of task demand. Given the participants’ language proficiency reflected in their responses to the SCICASA, we selected one section for L2 learners with an intermediate level of language proficiency from the database for TOEFL iBT integrated speaking. To address RQ2 which concerns the relationship between Chinese EFL learners’ metacognitive strategy use and the variability in task demands, we used all of the four different tasks in the section, and to ensure task validity, we did not make any modifications on the four tasks selected.

In the section, Task 1 presents a reading passage on a university’s new plan for shuttle route change, which is followed by a discussion between two university students on the plan in the listening section. After that, task-takers are required to state one of the speakers’ opinions on the new change. Task 2 provides a reading passage on a psychological concept: audience effect. In the following listening material, a lecture on this topic is delivered, and task-takers are asked to use the examples given in the listening section to explain the concept in the reading material. Task 3 involves a conversation between a professor and a female student on time conflict. To solve the conflict, the professor offers the female student two possible solutions, with neither sounding satisfactory to her. Task-takers are required to recommend one specific solution to the conflict and give the reasons why they believe such a solution might work. Task 4 is a lecture on two definitions of money in the listening section. The broad definition refers to both bills and the barter system. The narrow definition indicates the legal tender or whatever is accepted as payment such as coins in a society. Task-takers are asked to explain the two forms of money with the examples used by the professor in the lecture. Time for preparation before speaking is different, with 30 s for Task 1 and Task 2 and 20 s for Task 3 and Task 4. The varying degrees of task demand involved in the four tasks are displayed in Table 1.

**TABLE 1:** Variability in task demands in the four integrated speaking test tasks.

Tasks	Preparation time	Topic content	Language skills	Task type
Task 1	30 s	Campus-life situation	R-L-S	Opinion narrating
Task 2	30 s	Academic lectures	R-L-S	Concept-illustrating
Task 3	20 s	Campus-life situation	L-S	Problem-solving
Task 4	20 s	Academic lectures	L-S	Concept-illustrating

*s, seconds; R, reading; L, listening; S, speaking.*

#### TOEFL Integrated Speaking Test Rubrics

The TOEFL iBT integrated speaking rubric developed by the Educational Testing Service in 2008 was used by the two raters in scoring L2 learners’ performance. The rubric accommodates four criteria: delivery denoted by fluency, clarity, and pronunciation; language use referring to grammatical accuracy and vocabulary use; topic development indicated by cohesion and progression of ideas, and general description (Huang et al., 2018).

### Procedures

#### Data Collection

Chinese EFL learners answered the SCICASA each time they finished one integrated speaking test task. An electronic inventory in the form of word documents was delivered to the learners through a Chinese online survey system named “WenJuanXing”,^1^ which allowed them to use mobile phones for convenience and for research efficiency. Data collected on the system were automatically saved for our data analysis later. Data collection on the SCICASA for each Chinese EFL learner lasted around 10–20 mins. Chinese EFL learners performed the integrated speaking test tasks on computers with database software packages for TOEFL iBT integrated speaking. The learners’ responses to the speaking test tasks were recorded and stored automatically by the software packages as a single file. These files were then named after the learners’ codes. The order of those recording files was randomized using a random list generated in Microsoft Excel before they were given to the two raters. All the recording files were backed up in case of data loss (Weir et al., 2006).

By means of analytic scoring before holistic scoring, the two raters first scored independently the four segments of each Chinese EFL learner’s responses by referring to the rubric. A score ranging from 0 to 4 points was given to the four segments. Subsequently, the four scores were aggregated to form a composite score for each learner’s response to each task. The composite scores from the two raters for each response were then aggregated before they were divided to generate an average score which was used as the holistic score to measure the learners’ speaking performance statistically (Huang and Hung, 2013).

#### Data Analysis

Data preparation yielded 95 valid samples from the initial sampling of 134 participants, and the sample size meets the requirements of statistical testing methods involved in our study, including descriptive analysis, one-way repeated measures MANOVA, ANOVA, and the multiple regression analyses (Pallant, 2016; Frey, 2018). Following some scholars (e.g., Barkaoui et al., 2013; Sun, 2020, 2022a,b), we run a descriptive analysis of the means of L2 learners’ use of the four metacognitive strategies across tasks. We then used the line chart generated in Excel *via* the value of the means to illustrate the variance in the Chinese EFL learners’ IDs in metacognitive strategy use across tasks. The value of these means in combination with the chart was to address RQ1. Likewise, we used the means of the learners’ test scores to represent their speaking performance. To ensure scoring validity, inter-rater reliability was inspected with reference to Cronbach’s alpha coefficient. The index was 0.91, larger than the cutoff criterion (>0.70). This suggests the statistical validity of the scores rated by the two raters (Frey, 2018; Sun, 2020).

The subsequent data analysis for answering RQ2 was parsed into three steps. Step one targeted the relationship between Chinese EFL learners’ IDs in metacognitive strategy use and task demands involved in the four TOEFL iBT integrated speaking tasks within Kormos’ Model. In Step two, we investigated the relationships between the Chinese EFL learners’ IDs in metacognitive strategy use and their speaking performance. In Step three, we integrated the results in Step one with those in Step two to answer RQ2. In Step one, one-way repeated measures MANOVA was used, as the variable of IDs in metacognitive strategy use had four individual components and the variable of task demand had four task conditions represented by the four TOEFL iBT tasks. To run the one-way repeated measures MANOVA, a new variable that combined the four individual metacognitive strategies linearly was created to investigate the within-subject variance in the Chinese EFL learners’ reported use of the clustering metacognitive strategies across tasks. For identifying variance, values of F (*p* < 0.05) and η^2^ were examined, and the rule of thumb-up for these indices was as follows: If η^2^ is ≤0.01, it suggests a small effect size; a value ranging from 0.01 to 0.06 indicates a moderate effect size, and if η^2^ is ≥0.14, it indicates a larger effect size. The exact location of the variance in the four individual metacognitive components was further detected *via* the subsequent rounds of ANOVA, which followed the similar above data analysis principle of MANOVA (Pallant, 2016; Frey, 2018).

In Step two, we deployed the statistical procedure of multiple linear regression to assess how the four individual metacognitive strategies clustered to explain the Chinese EFL learners’ speaking performance while examining the associations between individual metacognitive strategies and speaking performance. The four subcomponents of the metacognitive strategies were entered into a model simultaneously as the predictor variables, and the Chinese EFL learners’ test scores were entered into the model as the outcome variable. Correlation coefficients (*r*) within the four individual metacognitive strategies were examined first for the appropriateness of the statistical procedure, and for inspecting multicollinearity: When *r* is ≤0.8, the employment of the procedure is suitable. Index regarding model fit was the adjusted *R*^2^, and the rule of thumb for the index is presented as the following:

<0.1: poor fit.

0.11–0.3: modest fit.

0.31–0.5: moderate fit.

>0.5: strong fit.

In addition, as the four strategies were measured on the same units on the SCICASA, the unstandardized coefficients (β) were inspected to investigate the impact of each individual metacognitive strategy on the Chinese EFL learners’ speaking performance. The cutoff *p*-value for β parameters is <0.01, indicating substantive effects of a specific metacognitive strategy on the learners’ speaking performance (Pallant, 2016; Frey, 2018). Finally, in Step three, based on the results generated in Step one and Step two, we examined the relationship in Chinese EFL learners’ IDs in metacognitive strategy use, task demand, and their speaking performance to answer RQ2.

## Results

### Chinese English-as-Foreign-Language Learners’ Individual Differences in Metacognitive Strategy Use Across Tasks for RQ1

**FIGURE 2:**
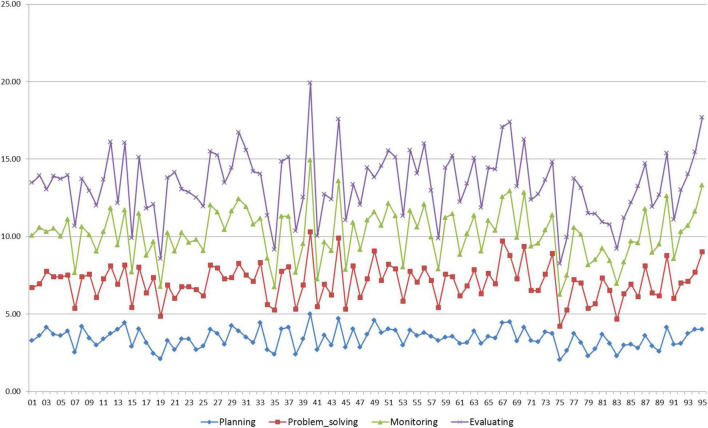
Variance in individual differences (IDs) in metacognitive strategy use among Chinese English-as-foreign-language (EFL) learners.

Figure 2 displays the variance manifested in the frequency of the 95 Chinese EFL learners’ use of planning, problem-solving, monitoring, and evaluating across the four TOEFL iBT integrated speaking tasks.

Table 2, on the other hand, revealed the descriptive statistics of the frequency by presenting the average means of the four individual metacognitive strategies across the four tasks. It is clear that problem-solving was reported by the Chinese EFL learners as the most frequently used, followed by planning and evaluating, while monitoring was the least frequently used strategy.

**TABLE 2:** Means of individual metacognitive strategies across tasks.

Tasks	Planning	Problem-solving	Monitoring	Evaluating
Task 1	3.61	3.90	3.17	3.17
Task 2	3.38	3.45	3.21	3.22
Task 3	3.53	3.69	3.18	3.36
Task 4	3.55	3.74	3.30	3.26
Average	3.52	3.70	3.22	3.26

The above results addressed **RQ1**: In the context of integrated L2 speaking assessment tasks within Kormos’ Model, Chinese EFL speakers’ use of the IDs construct of metacognitive strategies displayed variability, and among the four metacognitive strategies under investigation, problem-solving was used by the Chinese EFL speakers the most frequently, which was followed by planning, evaluating, and monitoring.

### Chinese English-as-Foreign-Language Learners’ Individual Differences in Metacognitive Strategy Use, Task Demand, and Performance for RQ2

#### Step One: Individual Differences in Metacognitive Strategy Use and Task Demand

In Step one that targeted the relationship between Chinese EFL learners’ IDs in metacognitive strategy use and task demand, with reference to the assumption test results for MANOVA, we used the indices of Pillai’s trace for the correction test. The more robust Pillai’s trace indices pointed out that there was a significant within-subject difference across task demands on the combined dependent variables or the Chinese EFL learners’ reported use of the clustering metacognitive strategies: F(12, 1212) = 12, *p* = 0.01 (less than the threshold of 0.05), and partial eta squared (η^2^) = 0.02. The result demonstrated a significant difference in the synergetic effect of task demands on the clustering metacognitive strategies in the Chinese EFL learners’ performance across tasks (Pallant, 2016; Frey, 2018).

To further locate the diffidence in the four individual metacognitive strategies across tasks, a series of separate ANOVAs were conducted. Each ANOVA was evaluated at an alpha level of 0.25 with Bonferroni adjustment. Results displayed that Chinese EFL learners’ reported use of problem-solving demonstrated modest heterogeneity across tasks [F (3, 405) = 3.85, *p* = 0.01, η^2^ = 0.02], whereas substantial variations were not found in the other three individual metacognitive strategies: planning [F(3, 405) = 1.21, *p* = 0.38, η^2^ = 0.01], monitoring [F(3, 405) = 0.42, *p* = 0.74, η^2^ = 0.003], and evaluating [F(3, 405) = 0.730, *p* = 0.47, η^2^ = 0.01] (Pallant, 2016; Frey, 2018).

#### Step Two: Individual Differences in Metacognitive Strategy Use and Speaking Performance

Step two focused on the relationships between Chinese EFL learners’ IDs in metacognitive strategy use and their speaking performance indicated by their oral scores. As displayed in Table 3, with reference to Table 1, which illustrates the varying task demands in the four tasks, the means of the Chinese EFL learners’ oral scores are as follows: Task 1 (narrating the speakers’ opinion on the university’s new policy) had the highest value, followed by Task 4 (illustrating a concept on money) and Task 2 (illustrating a concept on audience effect) in contrast to Task 3 (selecting a solution to time conflict), which ranked the lowest.

**TABLE 3:** Descriptive analysis of oral scores across tasks.

Tasks	Means	SD
Task 1	5.45	2.65
Task 2	4.40	3.15
Task 3	3.51	3.15
Task 4	4.86	2.99

Results of the subsequent multiple linear regression analysis showed that there were no significant interactive and individual effects of the four metacognitive strategies reported by Chinese EFL learners on their oral scores across tasks.

As shown in Table 4, values of the adjusted *R*^2^ on the four tasks were less than 0.1, suggesting a poor model fit. Alternatively stated, the four clustering metacognitive strategies explained a little in the variance of the Chinese EFL learners’ oral scores across the tasks. In addition, the *p*-values of the four tasks were all larger than 0.05, indicating that the four models built on the dataset of the four tasks were not the significant predictors of these learners’ speaking performance across tasks. The results implied that no substantial effects of the clustering metacognitive strategies on the Chinese EFL learners’ speaking performance across tasks were discovered.

**TABLE 4:** Relationship between the clustering metacognitive strategies and speaking performance across tasks.

Tasks	Adjusted *R*^2^	*df*	*F*	Sig.
Task 1	–0.36	4	0.18	0.95
Task 2	–0.00	4	0.86	0.49
Task 3	0.01	4	1.27	0.29
Task 4	0.01	4	1.19	0.32

Furthermore, Table 5 reveals that all the *p*-values of the β coefficients for the four subcomponents of the metacognitive strategies on the four test tasks were larger than 0.01. Such results revealed that the four individual metacognitive strategies had no relationships with the Chinese EFL learners’ speaking performance across tasks.

**TABLE 5:** Relationships between individual metacognitive strategies and speaking performance across tasks.

Tasks	Metacognitive strategies	β	*t*	Sig.
Task 1	Planning	0.06	0.22	0.83
	Problem-solving	0.26	0.65	0.52
	Evaluating	0.01	0.13	0.90
	Monitoring	–0.11	–0.27	0.79
Task 2	Planning	0.11	0.67	0.50
	Problem-solving	0.03	0.29	0.77
	Evaluating	0.15	1.11	0.27
	Monitoring	–0.09	–0.53	0.60
Task 3	Planning	–0.02	–0.04	0.97
	Problem-solving	0.95	2.16	0.03
	Evaluating	–0.71	–1.22	0.23
	Monitoring	0.19	0.41	0.68
Task 4	Planning	0.90	1.83	0.070
	Problem-solving	–0.27	–0.55	0.587
	Evaluating	0.30	0.66	0.513
	Monitoring	–0.62	–1.26	0.210

#### Step Three: Individual Differences in Metacognitive Strategy Use, Task Demand, and Performance

By integrating the results from Step one into those from Step two, we answered RQ2: planning, problem-solving, monitoring, and evaluating worked interactively, responding to task demands in the four TOEFL iBT integrated speaking tasks; the four metacognitive strategies, in individual and interactive working modes, had no significant effects on speaking performance.

## Discussion

### Chinese English-as-Foreign-Language Learners’ Individual Differences in Metacognitive Strategy Use Across Tasks

As revealed by the descriptive analysis shown in Figure 2, Chinese EFL learners’ use of metacognitive strategies differed from one another. This lends empirical evidence to the literature reviewed previously on the concept of IDs, which proposes metacognitive strategy use as one variable accounting for the concept (e.g., Griffiths and Soruç, 2021). Additionally, among the four metacognitive strategies under investigation, problem-solving was reported by Chinese EFL learners as the strategy they used most frequently. Such a result may have to do with how L2 learners performed the integrated speaking testing tasks. According to O’Malley and Chamot (1990), L2 learners tend to use strategies in a problem-solving manner, so it is possible that the Chinese EFL learners in our study considered their use of various strategies as an application of the problem-solving strategy and reported them on the inventory.

Indeed, in line with some scholars (e.g., Flavell, 1979; Zhang, 1999), L2 learners’ understanding of problem-solving strategy use reflects their metacognitive knowledge of strategies. As L2 learners’ metacognitive knowledge may be fallible or false, it is likely that they believe that they use the problem-solving strategy in performing tasks given, but in fact, they do not use such a metacognitive strategy at all. This may be true with the Chinese EFL learners in our study, which further explains the highest frequency of problem-solving use reported by them (Brown, 1987). The fallibility related to the Chinese EFL learners has been documented by Zhang (1999) whose study revealed the fallibility of Chinese university EFL learners’ metacognitive knowledge associated with their reading strategies.

Another possible reason for the highest frequency of the problem-solving strategy use may relate to L2 speech production. As reviewed earlier, in Kormos’ Model, unlike the other three metacognitive strategies which either work in a specific stage of the L2 speech process such as planning in conceptualization or work in a covert way during the process such as monitoring, problem-solving operates overtly throughout in L2 speech production, assisting L2 speakers to solve all the possible problems they might encounter in the speaking process. This “throughout” and “overt” characteristic is very likely to result in the highest frequency of problem-solving use reported by Chinese EFL learners in processing L2 speaking tasks.

In contrast, monitoring was reported as the least frequently used metacognitive strategy. This result is possibly due to L2 speech production. In Kormos’ Model, monitoring engages in speaking in both covert and overt manners. As the Chinese EFL learners had no prior knowledge of the four metacognitive strategies reported in our initial preparatory survey before the study, it is quite likely that they might not be able to identify monitoring when the strategy was working in a covert manner even though they were using it in the actual task performance. The lowest frequency of monitoring use in the integrated L2 speech assessment has borrowed some support from Swain et al. (2009), Yi (2012), and Barkaoui et al. (2013), the three studies that bear the closest relevance to this research, where metacognitive monitoring was found to be either not used at all or used the least frequently. Since monitoring works in tandem with evaluation as delineated afore, the low frequency of monitoring understandably contributed to the low frequency of evaluation, as was the case with this study.

Finally, the low frequency of planning use may be caused by Chinese EFL learners’ lack of prior knowledge of metacognitive strategy use discussed above: When individuals know nothing about how to use metacognitive strategies, it is understandable that they may not have the awareness of using these strategies in performing tasks. Such a relationship between one’s knowledge of metacognitive strategies and their use of these strategies has been reported elsewhere (e.g., Fazilatfar, 2010). The lack of motivation is likely to be another cause. According to some scholars (Oxford, 2017; Cohen, 2018), motivation is one of the most important individual factors that affect L2 learners’ strategy use, including planning. In this study, because Chinese EFL learners were volunteers and their performance did not affect their credit in the university, they might not be motivated enough to do systematic planning in performing the tasks given as proposed by Kormos and Wilby (2019) that learners’ task motivation considerably influences their strategy use.

### Chinese English-as-Foreign-Language Learners’ Individual Differences in Metacognitive Strategy Use, Task Demand, and Performance

#### Individual Differences in Metacognitive Strategy Use and Task Demand

In general, the slight statistical variance in Chinese EFL learners’ use of the individual metacognitive strategies across tasks illustrates the effect of task demands on these learners’ use of individual metacognitive strategies, though not substantial. The result coincides with the finding by Oxford et al. (2004), Swain et al. (2009), Yi (2012), and Barkaoui et al. (2013), in which the types and frequencies of the strategies used by participants were not found to be significantly affected by task demands. The reason for the loose correlation, as Barkaoui et al. (2013) have pointed out, has to do with the integrated L2 speaking tasks: Speaking tasks are highly demanding in terms of strategy use, so it is possible that L2 speakers use whatever metacognitive strategies at hand to tackle the speaking tasks in actuality. As a result, L2 speakers may not purposefully use strategies in response to a specific task demand.

Regarding the synergetic effects of the task demands on metacognitive strategy use, the high correlation index between the four individual metacognitive strategies and the output of the one-way repeated MANOVA suggest that these metacognitive strategies worked in an interactive manner reported by the Chinese EFL learners whose use of these strategies demonstrated substantial variability in response to the changing task demands. The result implies that metacognitive strategies operated in a clustering manner and they were task demand-dependent, which not only corroborates the working principle of IDs variables, including metacognitive strategies, that emphasizes the interactions between these variables with the external contexts or given tasks, but the working mode of metacognitive strategies as illustrated by Kormos’ Model and the literature on metacognition and LLSs as reviewed previously. Additionally, the result has been evidenced by a lot of literature on L2 assessment. For instance, Nett et al. (2012) whose longitudinal study with an experience sampling analysis on 70 German students showed that the metacognitive strategies used by the participants worked interactively in test performance. In addition, Fernandez (2018) unfolded the employment of the clusters of the metacognitive strategies reported in International English Language Testing System (IELTS) speaking tests *via* coding participants’ discourses. Moreover, the result concurs with Rukthong and Brunfaut’s (2020) study where metacognitive strategies were used concurrently by participants in their listening task performance.

#### Individual Differences in Metacognitive Strategy Use and Speaking Performance

Results of the multiple linear regression analysis indicate that generally, Chinese EFL learners’ use of metacognitive strategies, which worked individually and interactively, had no substantial effect on their speaking performance. Such results are not consistent with Kormos’ Model in which L2 speakers’ metacognitive strategy use is proposed to affect their final oral utterances. The reason for the inconsistency may have to do with the testing condition. Kormos’ Model is not formulated for the specific purpose of L2 speech production in testing conditions, while the L2 speaking context in our study is related to testing. It is known that under testing conditions, because of factors such as time limit that may cause learners’ anxiety and pressure, learners are unlikely to perform as well as they do under non-testing contexts in terms of strategy use (Huang et al., 2018). Consequently, it is possible that learners’ metacognitive strategy use displays no relationship with their speaking test performance, as discovered in our study. In fact, empirical research on the relationships between metacognitive strategy use and test performance, though extensive, has yet been inconclusive (e.g., Phakiti, 2016; Fernandez, 2018). In the current literature on L2 assessment, the weak relationship between L2 learners’ metacognitive strategy use and speaking test performance has been discovered in many studies. For example, in examining the relationship between individual metacognitive strategy use and test-takers’ integrated speaking test performance, Swain et al. (2009) and Barkaoui et al. (2013) found no significant and positive relations between the two variables. Similarly, Fernandez’s (2018) study showed no positive correlation between strategy use and participants’ test performance reflected by their test response quality in the IELTS speaking test tasks. These studies additionally lent some support to this study that resulted in a weak relationship between Chinese EFL learners’ metacognitive strategy use (either in a clustering manner or in an individual form) and their speaking test performance.

A possible alternative explanation is the instrument or the self-report inventory employed in our study to elicit Chinese EFL learners’ metacognitive strategies. Some researchers have pointed out that although self-report inventories have witnessed an extensive application in measuring metacognitive strategies, they may not represent what the participants actually do (e.g., Greene and Azevedo, 2010; Veenman and van Cleef, 2019). In accordance with this view, the metacognitive strategy use reported by the Chinese EFL learners on the SCICASA may not truly reflect their actual use of the strategies, and this may further affect the result, which we attained only through statistical analysis, on the relationship between the strategy use and the learners’ speaking performance.

## Conclusion and Implications

Although the results presented by our study were attained from only statistical methods, they will provide empirical evidence for validating Kormos’ (2006, 2011) Bilingual Speech Production Model and enrich the literature on the IDs in metacognitive strategy use. Simultaneously, the results will potentially add to the theory regarding the contextualization of Kormos’ Model in investigating the process of L2 speaking.

To start with, problem-solving was reported by Chinese EFL learners to be used most frequently in performing the integrated L2 speaking tasks. Such a report validates Kormos’ Model where L2 speakers resort to problem-solving to compensate for their incomplete L2 knowledge. Second, although an agreement has been reached on the role of metacognitive strategy use as a variable attributing to IDs, it is still unclear how IDs in metacognitive strategies work in actual language use situations. The salience of problem-solving in L2 speech production founded in our study obviously provides some insights into this research area: Among the various metacognitive strategies that are proposed to manifest IDs, problem-solving plays the most influential role in L2 speech production under authentic language testing conditions. Third, the disagreement between the weak relationship identified in this study concerning metacognitive strategy use and speaking performance within Kormos’ Model suggests that to understand IDs in metacognitive strategy use and its relationship with performance in L2 speech production within Kormos’ Model, it is necessary to take contexts into account: Are they testing contexts or non-testing contexts? It has been revealed by some studies as discussed earlier that under testing conditions, L2 speaker’s metacognitive strategy use may not display the sameness with that under non-testing conditions in which L2 speakers are unlikely to be bothered by test anxiety and pressure caused by testing conditions.

Our study into the concept of L2 speakers’ IDs manifested by their metacognitive strategy use and its relationships with task demands and performance has important implications for L2 speaking pedagogy and assessment. First, it indicates that in classroom instruction on metacognitive strategy use to perform L2 speaking tasks, in particular L2 speaking test tasks, teachers can set a special focus on the use of problem-solving since the highest frequency of the strategy has suggested that this strategy is easy for L2 speakers to reach and to use in dealing with L2 speaking test tasks. Such a teaching practice is in light of Oxford (2017), who proposed that EFL teachers’ attention be paid to students’ cognitive needs based on the students’ feedback on strategy use, including metacognitive strategy use. Furthermore, this teaching practice also corroborates Plonsky (2019), who supports a type of metacognitive strategy use instruction in classrooms where students tend to master the target strategies that are narrowed down by their teachers in the most effective way. Second, the relationship between L2 speakers’ use of metacognitive strategies and task demands involved in integrated L2 speaking tasks denotes that the holistic integrated L2 speaking tasks can effectively elicit L2 learners’ metacognitive strategy use and hence are proposed to be taken into consideration in teachers’ syllabus design or task development/design for the purpose of metacognitive strategy instruction on L2 speaking. This is essentially an answer to the call of some scholars (e.g., Frost et al., 2020; Zhang et al., 2021a,b) who advocate the inclusion of integrated skills tasks in classroom instruction for fostering language learners’ strategic competence, so that they can achieve learning sustainability. By the same token, regarding test developers, if they aim at testing test-takers’ metacognitive strategy use or strategic competence, integrated skill tasks are suggested to be taken into account in the process of test development (Bachman and Palmer, 2010). Finally, the weak relationship between metacognitive strategy use and speaking performance suggests that teachers purposefully create testing conditions in their classroom instruction on metacognitive strategy use by employing L2 speaking assessment/testing tasks. In this way, teachers can teach their students how to use metacognitive strategies effectively and efficiently in tackling testing conditions. Otherwise, if the teachers only teach their students’ metacognitive strategy use in non-testing conditions, the students may not know how to use metacognitive strategies in performing testing tasks, a type of tasks they usually take for getting a credit or for being enrolled by an institute (e.g., a university) of a higher level of education to achieve their academic success. As a result, the students may lose motivation in classroom instruction on metacognitive strategy use, which may further lead to the failures of the teachers’ pedagogical efforts (Zhang et al., 2021b).

## Limitations and Further Research

As delineated earlier, in this study, we only used self-report inventory to collect data. To increase the validity of self-report data, it is postulated that multiple procedures of data collection should be conducted (Creswell and Creswell, 2018). However, due to resource constraints, diverse means were not applied in our study, which may pose a threat to the validity of the research results. Moreover, as we used convenience sampling, the participants had similar backgrounds. Such sampling homogeneity may restrict the generalizability of the research results (Gurven, 2018). These limitations indicate that in future research of relevance diverse methods for data collection are suggested to be administered on heterogeneous sampling for improving the reliability and generalizability of the research findings.

## Data Availability Statement

The data are not publicly available due to ethical considerations. The raw data supporting the conclusions of this article will be made available upon request from the corresponding authors.

## Ethics Statement

The studies involving human participants were reviewed and approved by the University of Auckland Ethics Committee. The patients/participants provided their written informed consent to participate in this study.

## Author Contributions

WZ conceived of the initial idea, designed the study, collected and analyzed the data, and drafted the manuscript. MZ and YZ fine-tuned the design, and proofread the manuscript. All authors contributed to the article and approved the submitted version.

## Conflict of Interest

The authors declare that the research was conducted in the absence of any commercial or financial relationships that could be construed as a potential conflict of interest.

## Publisher’s Note

All claims expressed in this article are solely those of the authors and do not necessarily represent those of their affiliated organizations, or those of the publisher, the editors and the reviewers. Any product that may be evaluated in this article, or claim that may be made by its manufacturer, is not guaranteed or endorsed by the publisher.

## Footnotes

1
https://www.wjx.cn/index.aspx
